# Design of optically transparent metasurfaces based on CVD graphene for mmWave applications

**DOI:** 10.1038/s41598-023-31298-0

**Published:** 2023-03-25

**Authors:** Giovanni Magno, Lorenzo Caramia, Giuseppe Valerio Bianco, Giovanni Bruno, Antonella D’orazio, Marco Grande

**Affiliations:** 1 Polytechnic University of Bari, Bari, Italy; 2 CNR-NANOTEC, Bari, Italy

**Keywords:** Graphene, Electrical and electronic engineering

## Abstract

We propose and numerically investigate a smart, optically transparent digital metasurface reflective in the mmWave range, based on CVD graphene programmable elements. For both TM and TE polarizations, we detail the optimization of the unit cells, designed to exhibit two distinct states that correspond to those of binary encoding. The whole metasurface encoding can be customized to provide different electromagnetic functions, such as wide-band beam splitting at a controlled angle and reduction of the Radar Cross Section. Optically transparent metasurfaces could be integrated and exploited in windows and transparent surfaces in future Beyond-5G and 6G ecosystems.

## Introduction

Nowadays, the need for widespread and pervasive wireless connectivity is becoming more and more pressing as it underpins a flourishing amount of services and infrastructures^1^. Therefore, there is a need to focus research effort on integrating smart communication technologies into urban structures and building to meet the increasing bandwidth demands as well as environmental and aesthetic-architectural requirements. In this scenario, an ever-increasing number of users is in constant motion within a living urban environment which must become an active and synergic part of the ecosystem^2^. Indeed, a better service would be ensured by actively exploiting the surfaces available in the city environment in which users move. At the same time, smart surfaces must be able to dynamically adapt their function to the changing urban scenario. Nonetheless, their aesthetic appearance must be such as to seamlessly blend in with the cityscape. These considerations can also be extended to active indoor scenarios, where smart surfaces can be leveraged to improve the availability and effectiveness of services. For example, the increase in the average age of the world's population^3^ poses an urgent technological challenge aimed at mitigating and balancing the difficulties arising from the natural functional, physical, and cognitive decline that follows aging (slowing of psychomotor reflexes, reduced mobility, perception, memory, etc.). A huge benefit in this regard can be derived from services based on the location and navigation of people within their home environment. Traditional systems suffer from low accuracy in three-dimensional localization of subjects and high energy consumption. Intelligent reconfigurable metasurfaces, also known as Intelligent Walls, emerge as a promising alternative for multi-user localization^4–6^. Thus, obtaining smart, multifunctional, and reprogrammable (or even self-adaptively reprogrammable^7^) control of electromagnetic waves transmitted or reflected by a surface that can harmonize with the environment is of great interest for future generation wireless networks. This task may be accomplished by means of structured surfaces including material with tunable properties and may take advantage of the metasurfaces paradigm^8^. A metasurface is a quasi-2D artificially designed interface with tailored properties, typically achieved by adding a structuring in the wavelength scale or smaller^9^. Metasurface engineered properties, which has been proved to be attainable over an ultra-wide spectral range^10^, depend on materials, position, size, density, shape, and orientation of the structuring, which can be controlled by the designer to obtain a specific electromagnetic response that cannot be achieved by means of natural interfaces^11,12^. The action that such meta-structures imparts on an electromagnetic wave interacting with them is expressed in a strong control of both their "simple" properties (such as amplitude, phase and polarization) and their "exotic" ones (such as orbital properties, topological phase—Pancharatnam-Berry phase—and orbital momentum^13–16^). Their quasi-2D nature greatly simplifies the fabrication processes of these structures, reduces losses, eases integration with other devices^17^ and makes it possible to obtain exceptionally compact devices compared to traditional refractive elements. Finally, these structures can benefit from all the synergies well known in the metamaterials, photonic crystals and nanoantenna communities: exploitation of reconfigurable^18–21^ or active materials, inclusion of established paradigms such as transformation optics^22^, control of spatial dispersion^23–25^, guided resonances, etc.

A metasurface can be designed by exploiting Fermat's and Huygen's principles^26^. When an electromagnetic wave impinges on a surface, it induces electric and magnetic dipole moments, which, in turn, generate surface currents. According to Schelkunoff’s principle, these surface currents are equivalent to the tangential electric and magnetic fields which control the electromagnetic response of a surface. A metasurface can only be characterized as such only when each individual element is polarizable and subwavelength. This is only possible with proper design of the unit cell, which tailorizes its electromagnetic response as an elementary source. The superposition of these elementary contributions gives rise to the overall electromagnetic wavefront.

In this scenario, graphene constitutes the active element to achieve, at the same time, the desired metasurfaces tunability and transparency^8,21,27^. In fact, by modifying its chemical potential ${\mu }_{c}$, by means of chemical doping or electrical gating, its sheet resistance can be tuned, and, in turn, this modifies the unit cell response. It is worth stressing that graphene is not the only possible choice for designing optically transparent metasurfaces since there are other transparent conductive materials such as ITO. However, among the great advantages of using graphene are its extreme thinness (the 6-layer graphene has a total thickness of about 2 nm) and its flexibility and adaptability to curved surfaces. Conversely, the use of ITO, would result in much greater thicknesses (hundreds of nm) limiting the device to rigid and non-flexible substrates.

In this paper, we propose the design of a metasurface being optically transparent in the visible domain and behaving as a mirror in the mmWave domain. This metasurface integrates two graphene layers that enable static 1-bit digital programmability of its electromagnetic response.The paper is structured as follows: firstly, the general idea behind the proposed device is presented; then, the theory underpinning its operation is discussed; finally, design steps and numerical results are discussed.

## Proposed structure and design methods

### Proposed structure

**Figure 1:**
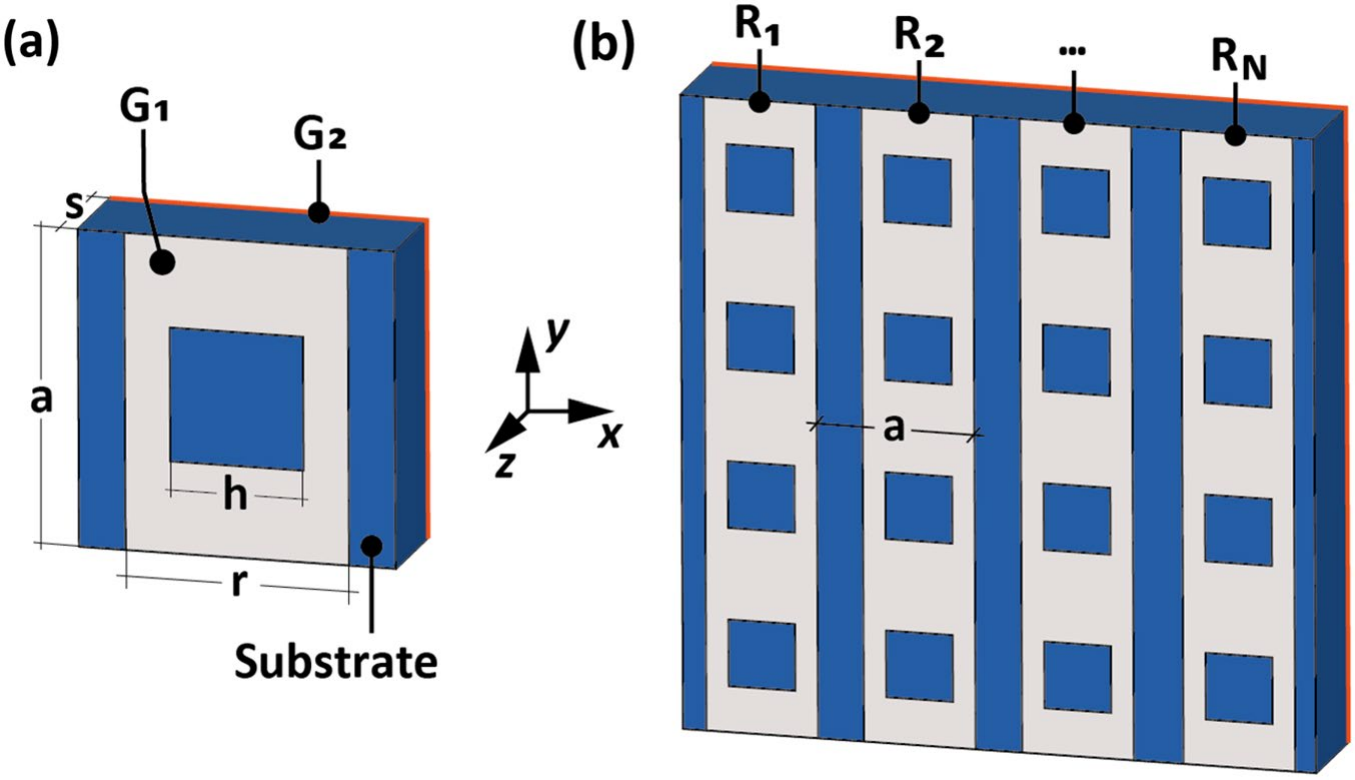
Sketch of the proposed geometry illustrating its structural composition and dimensions: (**a**) unit cell and (**b**) whole metasurface. Specifically, in (**b**) sheet resistance R_i_ value of each strip can be chosen independently from two possible values, R_s,0_ and R_s,1_.

Figure 1a shows the proposed optically transparent unit cell consisting of two graphene layers sandwiching a transparent dielectric substrate (with thickness *s* = 1.25 mm and a constant relative permittivity *ε*_*r*_ = 4.4). Specifically, several rectangular ring-shaped patterned graphene strips (constituting the layer G1) are placed on the top side of the dielectric substrate. G1 represents the programmable entity of the device. A uniform graphene layer (G2) is placed on the back side of the dielectric substrate and acts as a mirror. G1 is modelled as a graphene sheet with a variable 2D-ohmic sheet resistance equal to *R*_*s*_, while G2 is modelled as a SOCl_2_-doped six-layer graphene, with a constant sheet resistance equal to 20 Ω/sq and optical transparency of about 86.2%^28^. The square unit cell has a lattice constant *a* of 1.53 mm. As can be observed in Fig. 1a, rectangular rings (having a width *r* = 1.38 mm, a height equal to *a*, and a square hole of size *h* = 1.06 mm) are electrically continuous and discontinuous along the *y*- and *x*-directions, respectively. This allows setting the sheet resistance of each vertical strip of the metasurface to enable a 1-bit coding (the single bit addresses the whole vertical line, as in Fig. 1b). The proposed unit cell was numerically simulated via the Finite Element Method (CST Studio) for both TE and TM polarizations in Ka-band. The unit cell is excited through a plane wave at normal incidence. It is worth pointing out that the unit cell can be considered a one-port system since the transmittance is negligible due to the presence of the mirror layer G2^28^. In this case, the absorbance *A* is equal to 1-|*S*_*11*_(*R*_*s*_)|^2^^2^ in the mmWave spectral range, where *S*_*11*_(*R*_*s*_) represents the reflection coefficient as a function of the sheet resistance *R*_*s*_.

### Design of the unit cell

The unit cell design was carried out by identify two different values of the G1 sheet resistance, *R*_*s,0*_ and *R*_*s,1*_ associated to two different binary states, namely the ‘0’-state and the ‘1’-state, that must satisfy the following conditions:

1 $$\begin{array}{c}\left\{\begin{array}{l}\left|{S}_{11}\left({R}_{s,0}\right)\right|=\left|{S}_{11}\left({R}_{s,1}\right)\right| \\ |\Delta \phi |=|\phi \left({R}_{s,1}\right)-\phi \left({R}_{s,0}\right)|=\pi \end{array}\right.\end{array}$$

When Eq. (1) is satisfied, the two different states exhibit the same reflection coefficients |*S*_*11*_| and a π phase shift. In other words, the difference of the magnitudes of the reflection coefficients Δ|*S*_*11*_| =|*S*_*11*_(*R*_*s,1*_)|-|*S*_*11*_(*R*_*s,0*_)| must be zero and their phase difference Δ*ϕ* equal to 180°. It is worth noting that this approach can be generalized to obtain N-bit coding metasurfaces, where the discrete phase shift between contiguous states is equal to π/N. Henceforth, we will consider only 1-bit coding.

**Figure 2:**
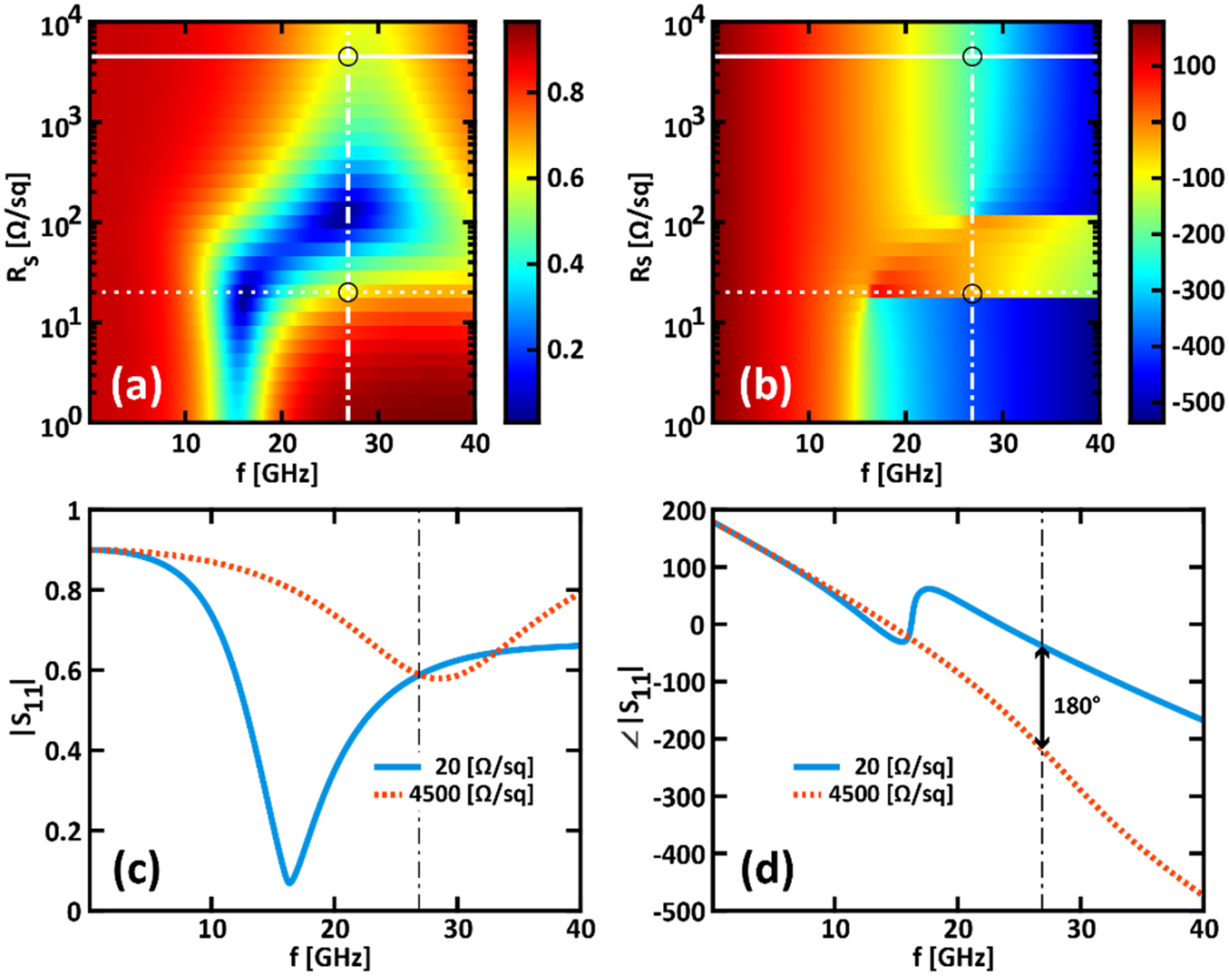
(**a**, **b**) Scattering parameter *S*_*11*_ (**a**) amplitude and (**b**) phase as a function of the frequency and of the sheet resistance varying from 10 Ω/sq to 10 kΩ/sq. The superimposed dashed and solid horizontal lines identify the sheet resistance values of 20 Ω/sq and 4500 Ω/sq, respectively. The vertical dotted dashed line indicates the frequency *f*_*0TM*_ = 26.87 GHz. Circular markers highlight the intersection between the superimposed horizontal and vertical lines. (**c**, **d**) Scattering parameter *S*_*11*_ (**c**) amplitude and (**d**) phase as a function of the frequency for *R*_*s*_ = 20 Ω/sq (solid cyan curves) and 4500 Ω/sq (dotted orange curves). (**a**–**d**) are obtained for a TM-polarized wave at normal incidence.

Figure 2 represents numerical results for the single unit cell excited by a TM-polarized plane wave impinging at normal incidence, within the spectral range of interest (1–40 GHz). In particular, Figs. 2a and b show the amplitude and phase of *S*_*11*_ as a function of the frequency. The dashed and solid horizontal lines identify two specific values of the sheet resistance (*R*_*s,0*_ = 20 Ω/sq and *R*_*s,1*_ = 4500 Ω/sq, respectively) for which the design equation Eq. (1) is satisfied at the frequency *f*_*0TM*_ = 26.87 GHz. This frequency is indicated by the vertical dotted dashed line, which intersects the horizontal lines at the points suggested by the circular markers. These two sheet resistance values embody the '0'- and '1'- states of binary encoding and will be considered from now on as associated with these states. Figures 2c and d show the amplitude and phase of *S*_*11*_ as a function of the frequency when the sheet resistance of the G1 layer is equal to *R*_*s,0*_ and *R*_*s,1*_, respectively. It is worth to point out that at *f*_*0TM*_ both states show the same amplitude of the reflected wave and a phase shift equal to 180°. Specifically, we found Δ|*S*_*11*_|≈− 0.04 dB (with |*S*_*11*_(*R*_*s,1*_)|≈ − 4.64 dB and |*S*_*11*_(*R*_*s,0*_)| ≈ − 4.60 dB). A sheet resistance value as low as 20 Ω/sq can be obtained through SOCl_2_ doping, as proved in ^28^, while a value of 4500 Ω/sq can be attained by means of hydrogenated graphene^28^. The number of graphene layers required to achieve these two sheet resistance values is equal to 6 and 1, leading to a total transparency of G1 of about 86.2% and 97.7%, respectively.

**Figure 3:**
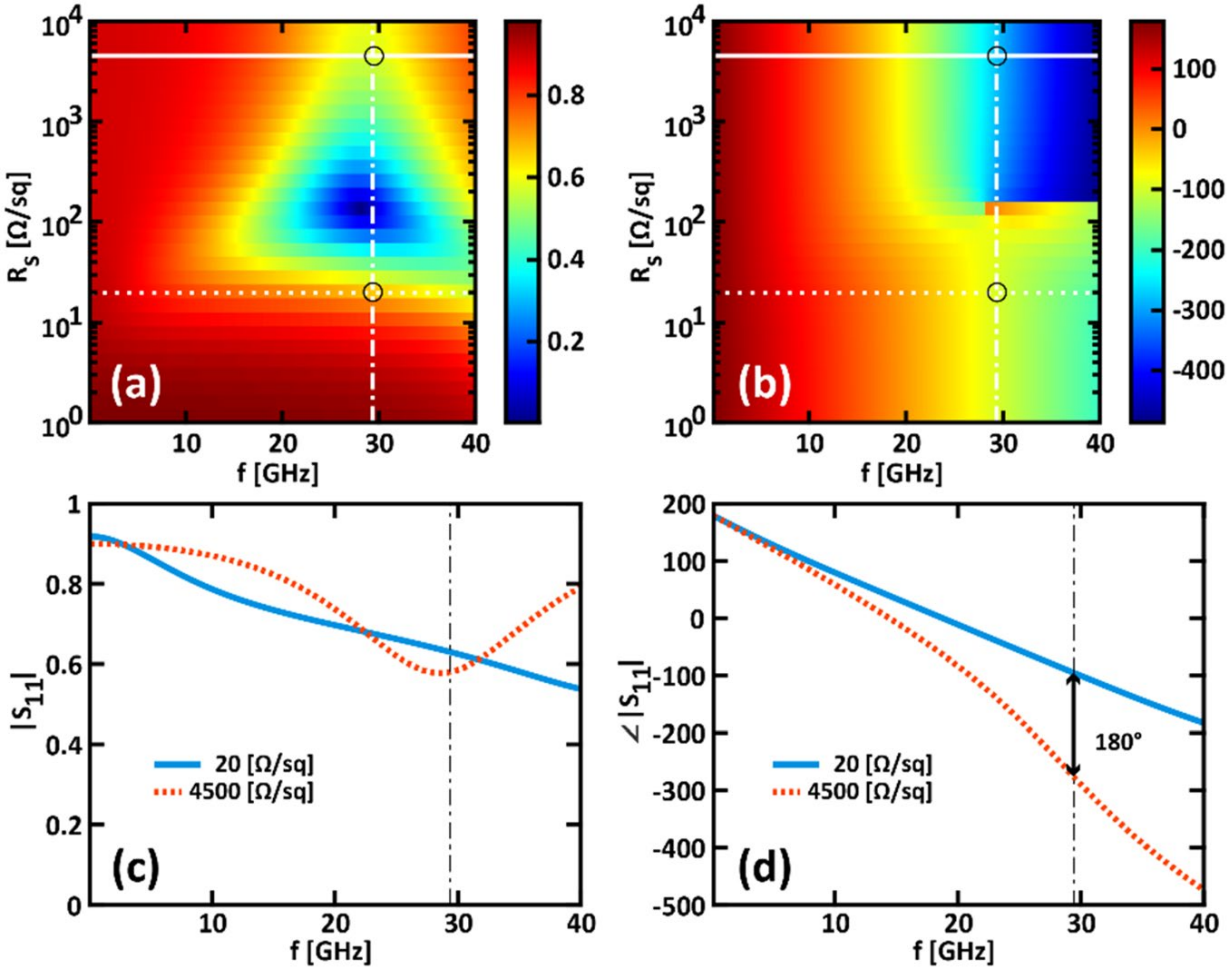
(**a**, **b**) Scattering parameter *S*_*11*_ (**a**) amplitude and (**b**) phase as a function of the frequency and of the sheet resistance varying from 10 Ω/sq to 10 kΩ/sq. The superimposed dashed and solid horizontal lines identify the sheet resistance values of 20 Ω/sq and 4500 Ω/sq, respectively. The vertical dotted dashed line indicates the frequency *f*_*0TM*_ = 26.87 GHz. Circular markers highlight the intersection between the superimposed horizontal and vertical lines. (**c**, **d**) Scattering parameter *S*_*11*_ (**c**) amplitude and (**d**) phase as a function of the frequency for *R*_*s*_ = 20 Ω/sq (solid cyan curves) and 4500 Ω/sq (dotted orange curves). (**a**–**d**) are obtained for a TE-polarized wave at normal incidence.

Similar results can be achieved for the TE-polarization, as shown in Fig. 3. In this case, Eq. (1) is quasi-satisfied at *f*_*0TE*_ = 29.39 GHz. Here, we found that |Δ*ϕ*|≈180° and Δ|*S*_*11*_|≈− 0.7 dB (with |*S*_*11*_(*R*_*s,1*_)|≈− 4.04 dB and |*S*_*11*_(*R*_*s,0*_)| ≈− 4.74 dB).

In Table 1 we show that, for these specific sheet resistance values, different phase shifts are observed by changing the frequency. For example, for the TM-polarized wave, by varying the working frequency around the design frequency between 25 and 28.5 GHz, we observe a change in the phase difference of ± 20° from the central value of 180°, and a change in the absolute value of the scattering coefficient Δ|*S*_*11*_| ranging from 0.003 to 0.071. Table 2 shows similar results for the TE-polarization.

**Table 1:** Values of |S_11_(f,R_s,0_)|, |S_11_(f,R_s,1_)|, Δ|S_11_| and |Δϕ| calculated for TM polarization and for different values of the working frequency.

Frequency [GHz]	|S_11_(f,R_s,0_)|	|S_11_(f,R_s,1_)|	Δ|*S*_*11*_|	|Δ*ϕ*| [°]
25	0.621	0.55	0.071	160
26	0.602	0.57	0.032	170
26.87	0.589	0.586	0.003	180
27.77	0.581	0.599	0.018	190
28.5	0.579	0.608	0.029	200

**Table 2:** Values of |S_11_(f,R_s,0_)|, |S_11_(f,R_s,1_)|, Δ|S_11_| and |Δϕ| calculated for TE polarization and for different values of the working frequency.

Frequency [GHz]	|S_11_(f,R_s,0_)|	|S_11_(f,R_s,1_)|	Δ|*S*_*11*_|	|Δ*ϕ*| [°]
28	0.578	0.639	0.061	160
28.7	0.577	0.634	0.057	170
29.39	0.580	0.628	0.048	180
30.1	0.586	0.622	0.036	190
30.8	0.595	0.616	0.021	200

### Design of the metasurface

When a finite arrangement of unit cells is considered, the overall radiation pattern of the metasurface *f*(*θ*,*ϕ*) can be written as:

2 $$\begin{array}{c}f\left(\vartheta ,\varphi \right)={f}_{e}\left(\vartheta ,\varphi \right)\sum_{m,n=0}^{N-1}{e}^{j\left[kd{\text{sin}}\left(\vartheta \right)\cdot \left(m{\text{cos}}\left(\phi \right)+n{\text{sin}}\left(\phi \right)\right)+{\varvec{\upxi}}\left(m,n\right)\right]}\end{array}$$

where *f*_*e*_(*θ*,*ϕ*) is the field radiated by the single unit cell, *θ* and *ϕ* are the elevation and azimuth coordinates, *N* is the number of unit cells in both *x* and *y* directions (a square metasurface is assumed), *d* is the period of the unit cells, *k* = 2π/*λ* is the wavenumber (where *λ* is the operating wavelength) and ***ξ*** is an *N* × *N* matrix containing the phase shift introduced by each unit cell.

The sum of Eq. (2) can be seen as the product of the array factors along the *x*- and *y*-directions:

3 $$\begin{array}{c}{\text{AF}}={\text{AF}}_{x}\cdot {\text{AF}}_{y}\end{array}$$

where

4 $${\text{AF}}_{x}=\sum_{m=0}^{N-1}{\text{exp}}\left\{j\left[kmd{\text{sin}}\left(\vartheta \right){\text{cos}}\left(\varphi \right)+{{\varvec{\xi}}}_{{\varvec{x}}}\right]\right\}$$

5 $${\text{AF}}_{y}=\sum_{n=0}^{N-1}{\text{exp}}\left\{j\left[knd{\text{sin}}\left(\vartheta \right){\text{sin}}\left(\varphi \right)+{{\varvec{\xi}}}_{{\varvec{y}}}\right]\right\}$$

and ***ξ***_***x***_ a and ***ξ***_***y***_ are N-elements arrays accounting for the phase shifts introduced by two adjacent elementary cells along *x*- and *y*-directions, respectively. Being the graphene strips G1 continuous along the *y*-direction, then ***ξ***_***y***_ = 0. Therefore, choosing a specific binary encoding of the metasurface means in practice deciding, during the fabrication process, the sequence of values of the sheet resistance of each graphene strip that constitutes the G1 layer between the values *R*_*s,0*_ and *R*_*s,1*_. This ultimately translates analytically into the choice of an appropriate ***ξ***_***x***_ array.

In the following paragraphs, we will exploit the previous equations to determine the overall response of the metasurface. In particular, we will vary the encoding of the metasurface to demonstrate different electromagnetic functions. On the one hand, we will show that by using “square wave” periodic encodings, the metasurface exhibits a beam splitting functionality with a controlled steering. On the other hand, in the presence aperiodic encodings, multiple secondary lobes may arise to help redistribute the reflected energy more uniformly in the available angular space. The latter behavior may be useful in reducing the Radar Cross Section (RCS).

## Results

In this section, the behavior of the metasurface as the encoding varies will be analyzed, when a plane wave impinging at normal incidence is considered. As a reference, the case of a constant encoding will be studied. Next, we will analyze the case of a "square wave" encoding, consisting of alternating equal numbers of '0' and '1' states, and the effect of period variation will be shown. Finally, the behavior of the metasurface in the presence of more irregular encodings will be analyzed.

Without loss of generality, from this point on, we will consider metasurfaces composed of 48 × 48-unit cells. The programming of each graphene strip constituting the G1 layer will be represented by a bit vector labeled ***C*** = ***ξ***_***x***_/π. Specifically, if the *n*-th element of the vector is 0 or 1, the *n*-th strip of G1 is programmed with a surface strength of *R*_*s,0*_ or *R*_*s,1*_, respectively. Only periodic encodings of length submultiple of N will be tested. To describe the encoding of the entire metasurface, we will use a bit subvector ***P*** of length *n*_*p*_ representing only the elements that constitute the period. Therefore, the overall encoding vector of the entire metasurface ***C*** is obtained by repeating *m* times the vector ***P***, such that *m* × *n*_*p*_ = *N*. In the case of a "square wave" encoding, the vector ***P*** will be denoted for convenience with compact notation as a pair of values ***P̃*** = (*Z*,*O*), where *Z* and *O* represent the number of consecutive '0' and '1' states constituting the period, respectively. For example, the periodic encodings i) ***P*** = (0,0,0,1,1,1), ii) ***P*** = (0,0,1,1), iii) ***P*** = (0,1) and iv) ***P*** = (1) or v) ***P*** = (0) will be represented as i) ***P̃*** = (3,3), ii) ***P̃*** = (2,2), iii) ***P̃*** = (1,1) and iv) ***P̃*** = (0,1) or v) ***P̃*** = (1,0) , respectively. Hence, in the case of *N* = 48, ***P̃*** = (0,1) and ***P̃*** = (1,0) represent metasurfaces in which the 48 graphene strips are uniformly programmed with '1' and '0' states, respectively.

**Figure 4:**
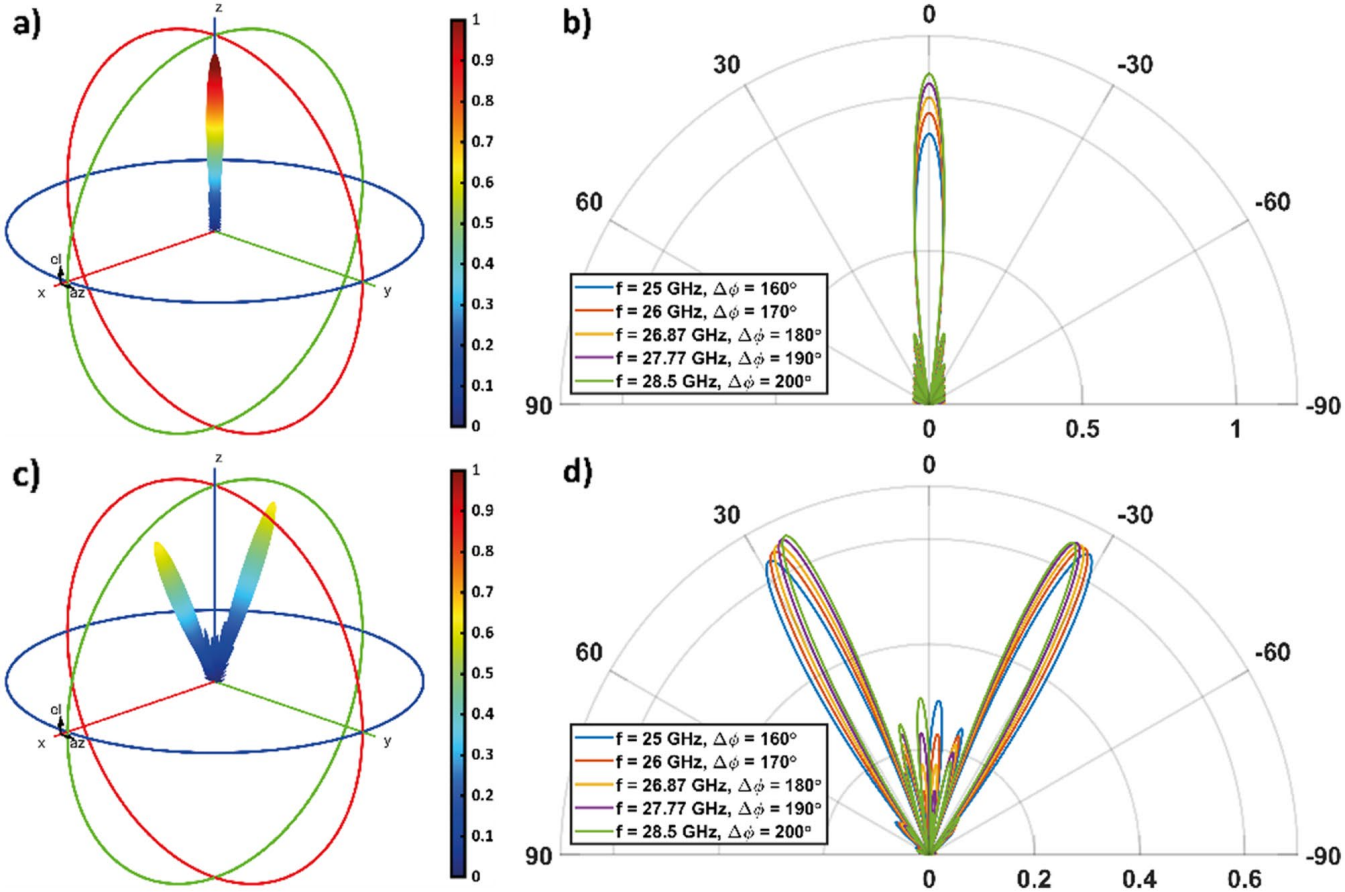
Overall radiation pattern of the metasurface when (**a**, **b**) **P** = (1) and (**c**, **d**) ***P̃*** = (8,8) is considered, when the the incident wave is TM-polarized. In (**a**) and (**c**) the 3D radiation pattern is shown for *f* = *f*_*0TM*_ = 26.87 GHz (∆*ϕ* = 180°). (**b**, **d**) 2D radiation pattern polar plot, calculate in the [x,z]-plane, for different frequency values.

As a reference case, we consider a constant metasurface encoding, in which the sheet resistances of each graphene strip of G1 are set equal to *R*_*s,1*_. This corresponds to a binary encoding ***P*** = (1). Figures 4a and b show the overall radiation pattern in the TM case, where we observe the presence of a single lobe in the reflected energy along the direction normal to the metasurface. When the frequency increases from 25 to 28.5 GHz, the maximum lobe intensity increases of about 1.78 dB, from about 0.88 to 1.08, while the side lobes remain lower than about 0.23.

Let us now consider a periodic, nonconstant encoding. In particular, we consider a "square wave" encoding, with a vector ***P̃*** = (8,8) of length equal to 16. The corresponding radiation pattern in the case of TM polarization is shown in Figs. 4c and d. We observe that this encoding allows the realization of a beam splitting function, where the energy of the single lobe observed in the case of constant encoding splits into two distinct quasi-symmetrical lobes (the quasi-symmetry reflects that of the encoding).

In particular, in Fig. 4d we observe that the main lobes intensity slightly increases as the frequency increases from 25 to 28.5 GHz and Δ*ϕ* increases from 160° to 180° (left main lobe: about 0.63 to 0.66; right main lobe: about 0.64 to 0.65). As well their spreading decreases by about 4° as Δ*ϕ* increases from 160° to 180° (when f is equal to 25, 26.87 and 28.5 GHz the left lobe is oriented at about 28.5°, 26.5° and 24.5°, respectively, while the right lobe is oriented at about − 28°, − 26°, and − 25°, respectively). Besides, side lobes become more intense as we deviate from the condition Δ*ϕ* = 180°. They reach about 44% of the main lobes at f = 25 GHz and f = 28.5 GHz, where we have Δ*ϕ* = 160° and Δ*ϕ* = 200°, respectively. This ratio drops to about 32% at *f* = *f*_*0TM*_ = 26.87 GHz when Δ*ϕ* = 180°.

**Figure 5:**
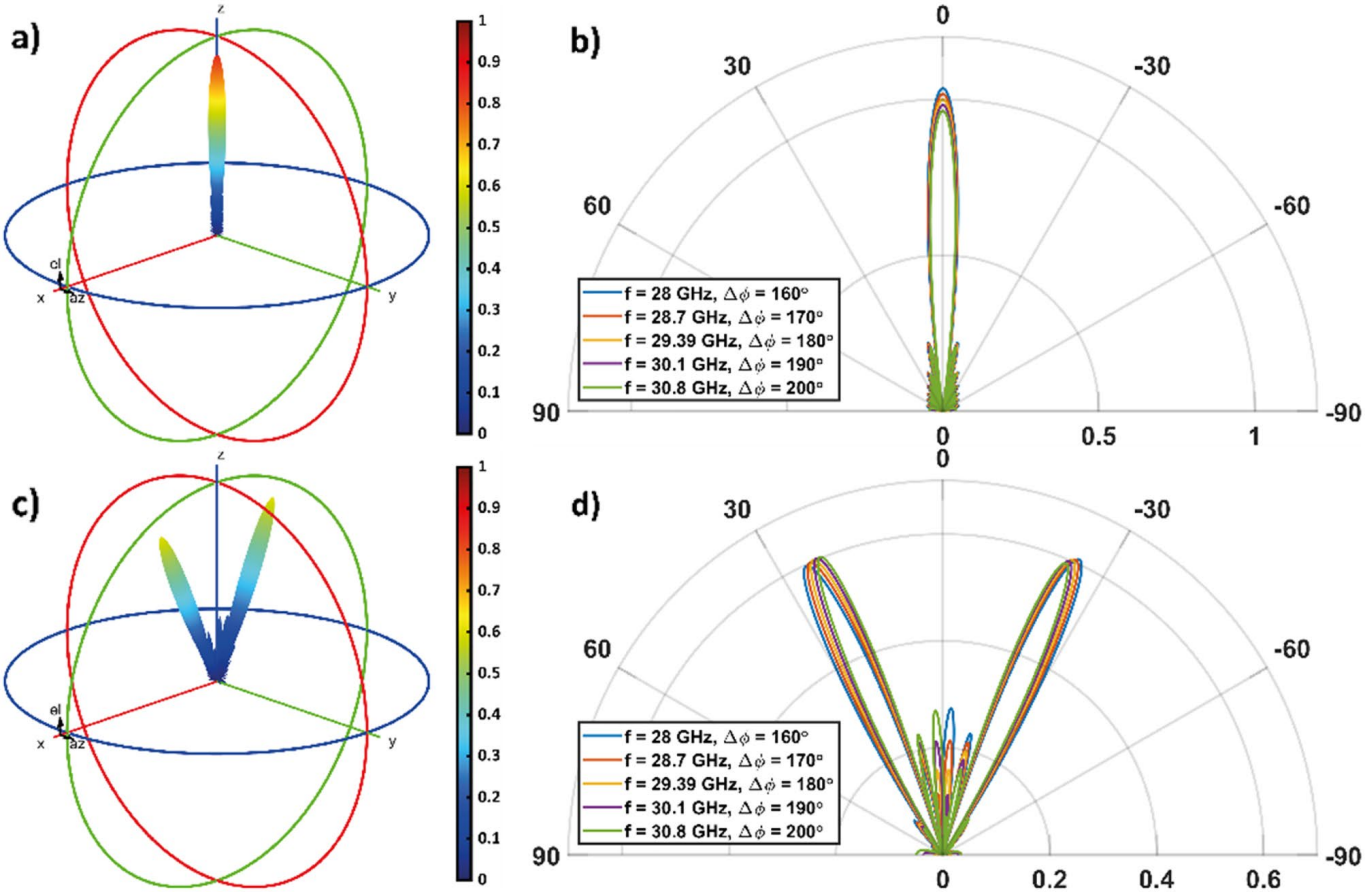
Overall radiation pattern of the metasurface when (**a**, **b**) **P** = (1) and (**c**, **d**) ***P̃*** = (8,8) is considered, when the the incident wave is TE-polarized. In (**a**) and (**c**) the 3D radiation pattern is shown for *f* = *f*_*0TE*_ = 29.39 GHz (Δ*ϕ* = 180°). (**b**, **d**) 2D radiation pattern polar plot, calculate in the [x, z]-plane, for different frequency values.

Figure 5 shows the 3D and 2D radiation patterns when an impinging wave with a TE-polarization is considered. If the metasurface is uniformly programmed with a ''1'' state, the results are similar to the TM case. The maximum amplitude is achieved at f = 28 GHz and, as the frequency increases, the amplitude decreases. The side lobes remain lower than about 0.22. Results for the coding pattern ***P̃*** = (8,8) in case of TE polarization are shown in Figs. 5c and d.

In particular, in Fig. 5d we observe that the main left (right) lobe intensity slightly increases (decreases) as the frequency increases from 25 to 28.5 GHz and Δ*ϕ* increases from 160° to 180° (left main lobe: about 0.59 to 0.60; right main lobe: about 0.61 to 0.59). As well their spreading decreases by about 3° as Δ*ϕ* increases from 160° to 180° (when f is equal to 28, 29.39 and 30.8 GHz the left lobe is oriented at about 25.4°, 23.7° and 22.4°, respectively, while the right lobe is oriented at about − 24.8°, − 23.7°, and  −22.9°, respectively). As for the TM case, here side lobes become more intense as we deviate from the condition Δ*ϕ* = 180°. They reach about 45% of the main lobes at *f* = 28 GHz and *f* = 30.8 GHz, where we have Δ*ϕ* = 160° and Δ*ϕ* = 200°, respectively. This ratio drops to about 33% at *f* = *f*_*0TE*_ = 29.39 GHz when Δ*ϕ* = 180°.

**Figure 6:**
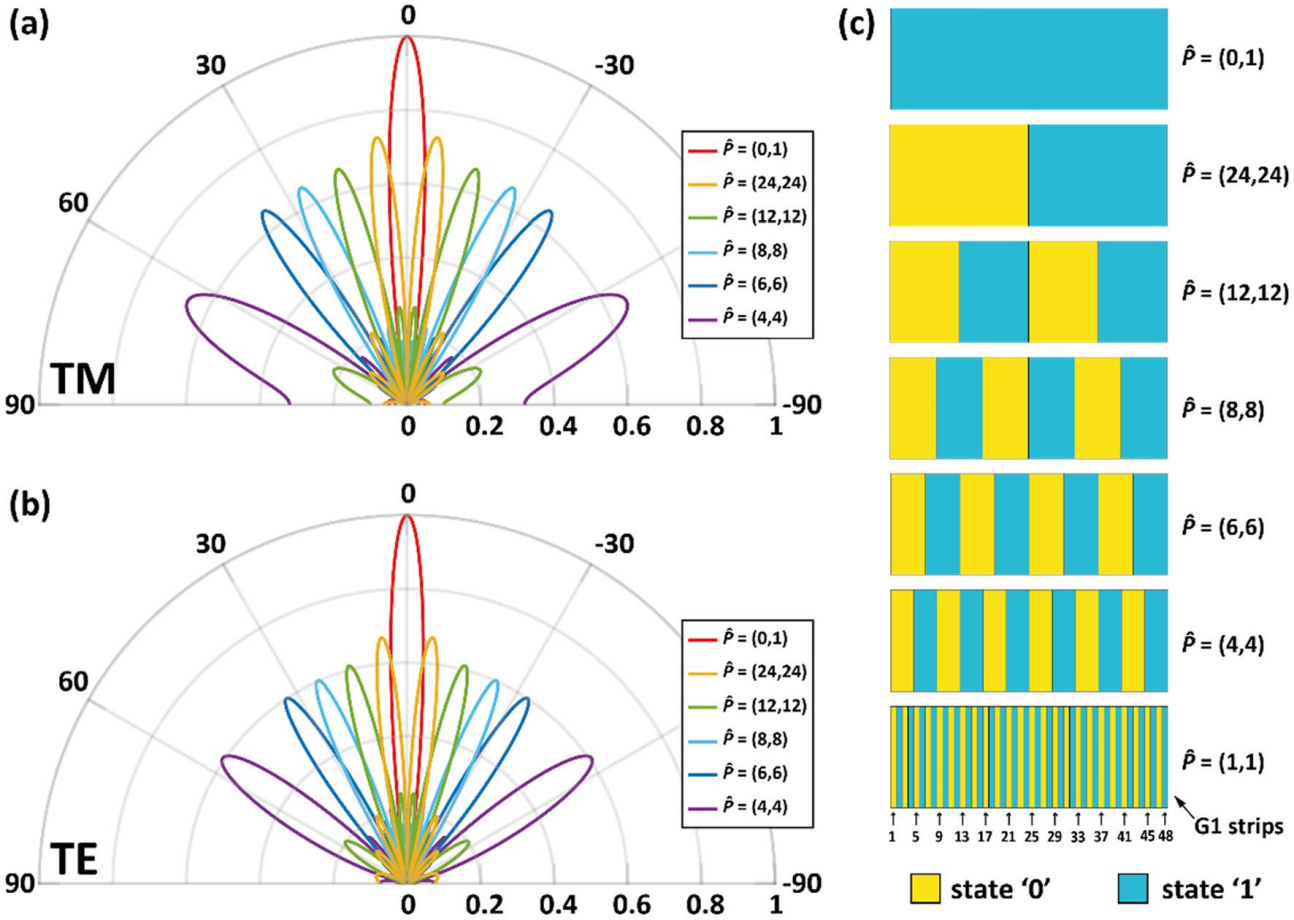
(**a**) TM and (**b**) TE overall radiation pattern of the metasurface when "square wave" encodings are considered. (**c**) Representation of the state of each G1 strip for encodings ***P̃*** = (0,1), ***P̃*** = (24,24), ***P̃*** = (12,12), ***P̃*** = (8,8), ***P̃*** = (6,6), ***P̃*** = (4,4) and ***P̃*** = (1,1).

**Figure 7:**
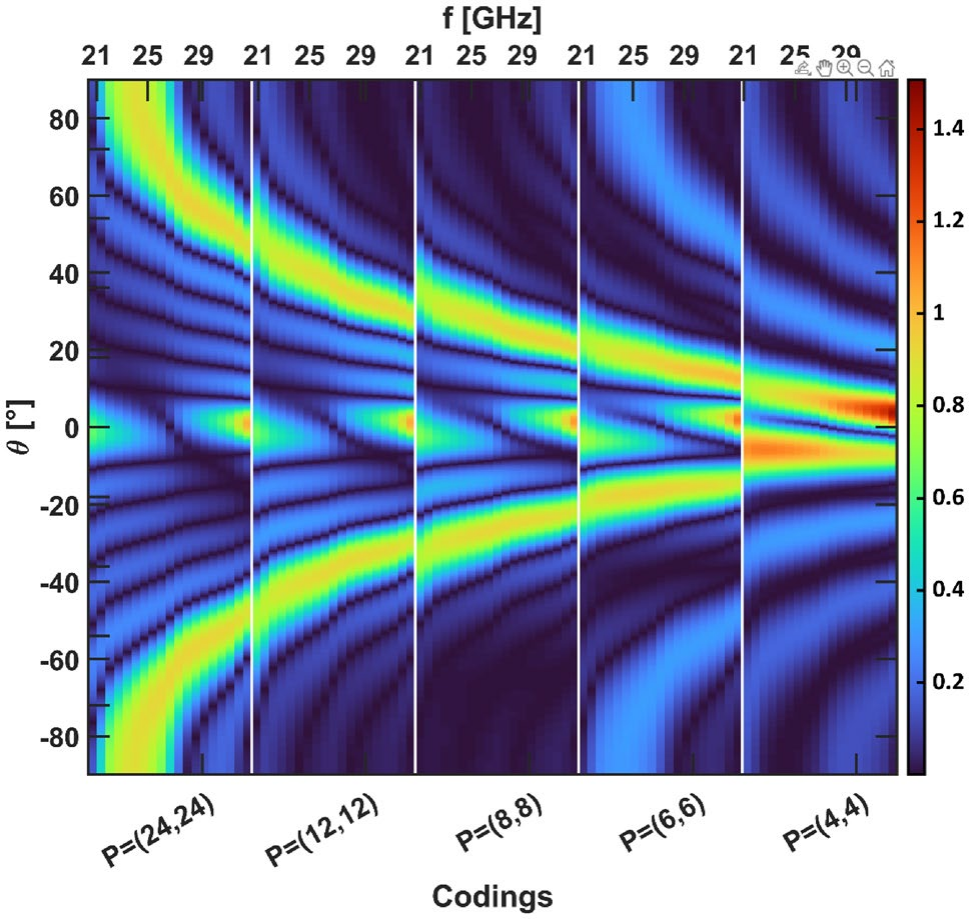
Radiation pattern of the metasurface as a function of the angle θ, of the encoding pattern (the different coding patterns are shown on the lower horizontal axis and segregated by vertical white lines), and of the frequency (shown on the upper horizontal axis, it recurs for each coding pattern) when the incident wave is TM-polarized.

We now analyze the effect of period length variation on the beam splitting function when considering "square wave" encodings. Figures 6a and b show the 2D radiation patterns for encodings with decreasing period, for both polarizations. Represented in Fig. 6c are the corresponding encodings ***P̃*** = (0,1) (reference constant ‘1’ state coding), ***P̃*** = (24,24) (the right (left) half of the metasurface is in the '0' ('1') state), ***P̃*** = (12,12), ***P̃*** = (8,8), ***P̃*** = (6,6), ***P̃*** = (4,4). The encoding ***P̃*** = (1,1) is also shown in Fig. 6c to help identifying the different graphene strips of G1 and their corresponding state. For both polarizations as the length of the repetition of the '0' and '1' states decreases, an increase in the separation angle of the beam splitting function and a slight decrease in the intensity of the beams are observed. Table 3 shows the intensity and elevation angle values of the one of the two symmetrical main lobes (the one located in the half-space for positive *θ*) for the above encodings. This analysis shows that by varying the length of the "square wave", that constitutes the encoding of the metasurface, it is possible to change the beam splitting angle over discrete values between 0° and 64.91° for TM polarization and between 0° and 55.91° for TE polarization. Moreover, it should be noted that it is possible to continuously change the beam splitting angle by tuning the working frequency around the design frequency, as discussed earlier. Besides, by simultaneously changing the working frequency and the coding, it is possible to achieve continuity of steering along almost the entire range of theta angle. Figure 7 shows the radiation pattern of the metasurface as a function of theta angle, encoding (which varies between P(24,24) to P(4,4), the different encodings are separated by a vertical white line) and frequency (which varies between 21 and 33 GHz and repeats with each change of encoding). In the figure we observe that the angles swept by the beams vary almost continuously along the entire observation space. Finally, to obtain steering at a fixed frequency, other non-periodic or pseudo-periodic encodings need to be investigated in future efforts to identify how finely the angle discretization can be reduced.

**Table 3:** Values of the intensity and elevation angle of the main lobe located in the half-space for positive *θ* as a function of metasurface encoding.

*P̃*	TM		TE	
	Value	Angle [°]	Value	Angle [°]
(0,1)	1.00	0.00	1.00	0.00
(24,24)	0.73	** ± **6.55	0.67	** ± **6.00
(12,12)	0.67	** ± **16.36	0.61	** ± **15.00
(8,8)	0.66	** ± **26.18	0.60	** ± **23.73
(6,6)	0.65	** ± **36.55	0.60	** ± **33.00
(4,4)	0.66	** ± **64.91	0.61	** ± **55.91

**Figure 8:**
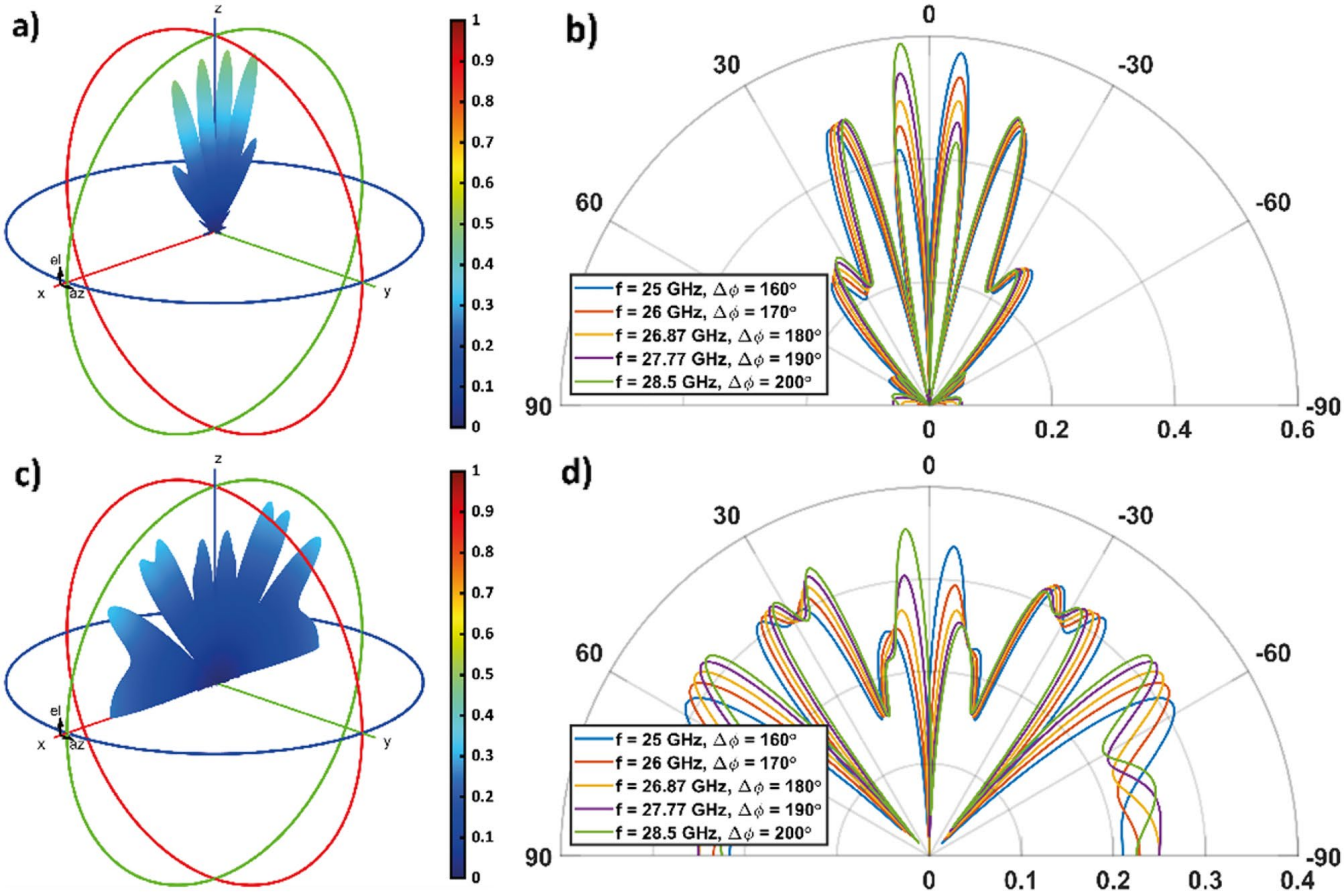
Overall radiation pattern of the metasurface when (**a**, **b**) ***Ĉ***_***8***_ = (0,0,1,0,1,1) and (**c**, **d**) ***Ĉ***_***4***_ = (0,0,1,0,0,1,1,1,0,1,0,1) is considered, when the incident wave is TM-polarized. In (**a**) and (**c**) the 3D radiation pattern is shown for *f* = *f*_*0TM*_ = 26.87 GHz (Δ*ϕ* = 180°). (**b**, **d**) 2D radiation pattern polar plot, calculate in the [x,z]-plane, for different frequency values.

**Figure 9:**
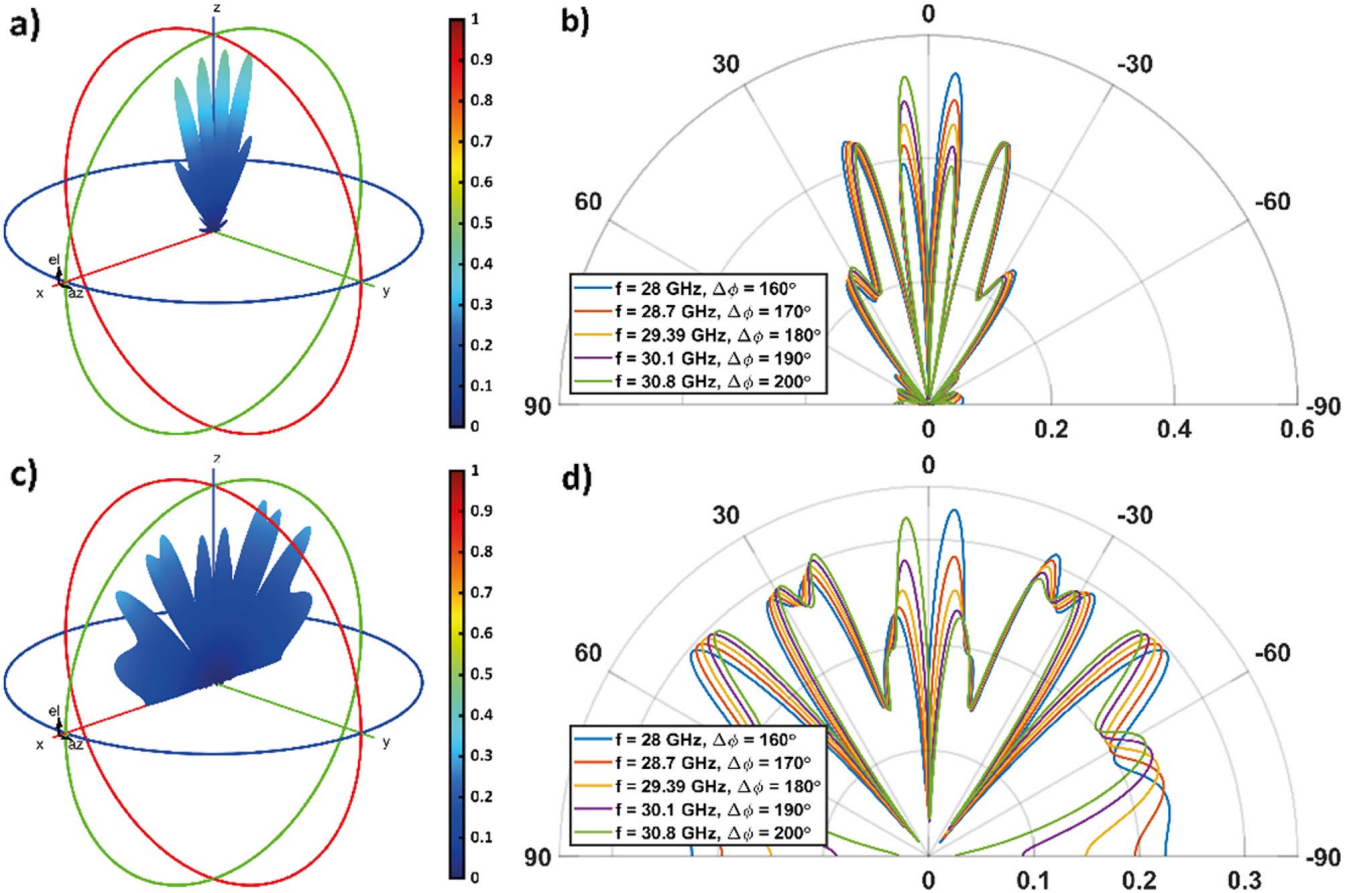
Overall radiation pattern of the metasurface when (**a**, **b**) ***Ĉ***_***8***_ = (0,0,1,0,1,1) and (**c**, **d**) ***Ĉ***_***4***_ = (0,0,1,0,0,1,1,1,0,1,0,1) is considered, when the incident wave is TE-polarized. In (**a**) and (**c**) the 3D radiation pattern is shown for *f* = *f*_*0TE*_ = 29.39 GHz (Δ*ϕ* = 180°). (**b**, **d**) 2D radiation pattern polar plot, calculate in the [x,z]-plane, for different frequency values.

For certain non-periodic coding patterns, the response of the metasurface becomes more disordered. New lobes appear that help to backscatter the incident energy more uniformly in all available directions. The function manifested by the metasurface may therefore resemble that of RCS reduction. Non-periodic coding patterns are conveniently describable with a different notation. We therefore introduce the vector ***Ĉ***_***M***_ = (*a*_*1*_,*a*_*2*_,..) as a compact representation of the vector ***C***, in which the subscript *M* is an integer representing the number of times each state *a*_*i*_ of ***Ĉ***_***M***_ must be repeated to obtain ***C***. For instance, ***Ĉ***_***2***_ = (0,1) and ***Ĉ***_***3***_ = (0,1,0) corresponds to ***C*** = (0,0,1,1) and ***C*** = (0,0,0,1,1,1,0,0,0), respectively. To show the aforesaid RCS reduction function, we consider the following two coding patterns, ***Ĉ***_***8***_ = (0,0,1,0,1,1) and ***Ĉ***_***4***_ = (0,0,1,0,0,1,1,1,0,1,0,1), which will be investigated first for TM polarization (results are presented in Fig. 8), then for TE polarization (results are presented in Fig. 9).

Figures 8a and b show the results for the coding pattern ***Ĉ***_***8***_ = (0,0,1,0,1,1) for the TM polarization. It is worth noting that, on the one hand, in the configurations operating at *f* = 25 GHz and *f* = 28.5 GHz, a major lobe with amplitude close to 0.6 is observed; while, on the other hand, when Δ*ϕ* tends to 180°, the major and minor lobes have comparable amplitudes (at the working frequency *f* = *f*_*0TM*_ = 26.87 GHz, the amplitude remains below 0.5). As the frequency changes, the lobe angles undergo minimal drift.

Figures 8c and d show the results for the coding pattern ***Ĉ***_***4***_ = (0,0,1,0,0,1,1,1,0,1,0,1), for TM polarization. Compared with the previous encoding, we observe from the radiation pattern that energy is reflected in a more spread-out fashion in all available angular directions. The amplitude of the reflected wave is always smaller than 0.36 in the spectral range of observation (*f* between 25 and 28.5 GHz).

Similar results are obtained in the case of the TE polarization, shown in Fig. 9, where a slight decrease in the amplitude of the radiation pattern can be seen for both analyzed encodings. In particular, this is maintained below 0.6 (coding pattern ***Ĉ***_***8***_ = (0,0,1,0,1,1), shown in Figs. 8a and b and 0.3 (coding pattern ***Ĉ***_***4***_ = (0,0,1,0,1,0,1,0,1), shown in Figs. 9c and d in the spectral range of observation. It is worth pointing out, as the size of the two-dimensional array increases, larger $M$ values may be exploited, and other coding sequences can be used to improve the performance of the structure in terms of RCS reduction.

## Conclusion

In this work, the design of a smart and optically transparent metasurface in mmWave range, based on CVD graphene, was proposed. Specifically, the unit cell was designed in terms of the amplitude and phase of reflection coefficient *S*_*11*_ to identify two distinct states, labeled '0' and '1,' that can mimic the behavior of binary encoding. In particular, for such states, metasurface reflection coefficient shows the same amplitude and a phase difference of π. This led to the identification of two possible values of the sheet resistance of the decorated graphene stripes that constitute the programmable layer of the metasurface and which can be chosen during the manufacturing phase to impart the desired electromagnetic response. The optimized unit cell shows an optically transparency of about 84%. We have also shown that as the encoding of the entire metasurface changes, different electromagnetic functions occur. We have demonstrated that, for both TE and TM polarizations, using periodic "square wave" encodings, a beam splitting function is observed with a beam separation angle that depends on the period length of the encoding. In the presence of aperiodic encodings, the arising multiple secondary lobes can help the uniform redistribution of reflected energy, which is useful in reducing the radar cross section (RCS). The proposed structure may be fabricated by combining (i) CVD graphene Reactive Ion Etching patterning (with a 3D-printed mask) and (ii) graphene transfer techniques^29^. In addition, by exploiting electrolytic gating to modify the graphene chemical potential, the device has the potential to improve its reconfigurability from statically programmable to dynamically tunable. The proposed device may be extended to flexible and conformal substrates and could be integrated with optical devices for the realization of active, hybrid systems devoted to B5G and 6G ecosystems.

## Data Availability

The datasets used and/or analysed during the current study available from the corresponding author on reasonable request.
